# Chronic Nitrous Oxide Toxicity Despite Elevated Serum Vitamin B_12_ Level: A Case Report

**DOI:** 10.5811/cpcem.39674

**Published:** 2025-02-08

**Authors:** Jessica Graves, John Hafner

**Affiliations:** * Mercy Health-Lourdes Hospital LLC, Department of Emergency Medicine, Paducah, Kentucky; † The University of Illinois College of Medicine, Department of Emergency Medicine, Peoria, Illinois

**Keywords:** case report, nitrous oxide toxicity, neurotoxicity, functional vitamin B_12_ deficiency

## Abstract

**Introduction:**

Nitrous oxide (N_2_O) toxicity is an uncommon but important-to-recognize presentation of neurologic deficits and hematologic abnormalities, which may never resolve in some patients. In the United States, nitrous oxide is legal to possess and easily obtainable for purchase in stores and online. Nitrous oxide abuse and its long-term sequelae must be recognized by the emergency physician to ensure proper follow-up and maximize neurologic outcomes.

**Case Report:**

A 28-year-old male with past medical history of alcoholism and polysubstance abuse presented to the emergency department with progressive paresthesias, shortness of breath, and neurologic complaints following daily inhalation of N_2_O for three weeks. He was diagnosed with N_2_O toxicity due to functional vitamin B_12_ deficiency in the setting of elevated B_12_ levels from prophylactic self-supplementation.

**Conclusion:**

While most recreational users of nitrous oxide will experience transient neurologic symptoms resolving within minutes of cessation, frequent or heavy users may develop permanent neurotoxicity. Exposed patients require close follow-up with neurology and vitamin B_12_ supplementation to maximize neurologic recovery. In this patient, there was persistence of neurologic symptoms over 24 hours after cessation of use despite self-supplementation of vitamin B_12_.

## INTRODUCTION

Nitrous oxide (N_2_O), also known as “laughing gas,” is an inhaled compound commonly used for medical and dental procedures for anesthesia and anxiolysis. It is also used commercially for food preservation, fuel combustion, and aerosolization of food products (such as whipped cream), making it widely available to purchase. Nitrous oxide is abused recreationally due to its euphoric side effects and hallucinogenic properties, and the prevalence of abuse has increased in recent years.^1^ The onset of effects occurs within seconds of inhalation. In the vast majority of users, the effects of N_2_O dissipate within minutes. However, prolonged use may create dose-dependent effects resulting in persistent neurologic deficits after cessation of the drug.

These neurologic deficits are related to the drug’s impact on the body’s utilization of vitamin B_12_, creating a functional B_12_ deficiency.^2^ The mainstay of treatment involves cessation of the drug and supplementation with vitamin B_12_. As the popularity of recreational use of N_2_O rises, online communities of users have recommended prophylactically supplementing B_12_ to its members; however, as demonstrated by this case report, prophylactic vitamin B_12_ supplementation may not prevent neurotoxicity.

## CASE REPORT

The patient was a 28-year-old male with a history of alcoholism, cocaine use, and marijuana use. He presented to the emergency department (ED) with a chief complaint of paresthesias and neurologic symptoms after prolonged use of recreational N_2_O. The patient reported buying a commercial-sized tank of N_2_O from an online retailer three weeks prior to presentation. He described inhaling the N_2_O repeatedly since obtaining it and discontinuing its use just over 24 hours prior to presentation to the ED. He reported inhaling directly from the tank an estimated at 30–60 times per hour during awake hours daily. In addition to the N_2_O use, he was also smoking marijuana daily and using cocaine and alcohol every few days.

He reported onset of progressive symptoms over the prior three to five days, consisting of numbness and tingling of the extremities, “brain fog,” headaches, visual hallucinations, chest pain, dyspnea, and nausea. He reported using inhaled N_2_O in the past without any prolonged symptoms. He was concerned that his symptoms were not resolving despite cessation of N_2_O use over 24 hours prior to presentation. He reported symmetric ascending paresthesias initially involving the lower extremities, which were now present to a lesser extent in the hands and wrists, described as tingling and numbness without motor deficits. He complained of a holocephalic, pressure-like headache and intermittent visual hallucinations described as “shadows” in his peripheral vision. He reported that he was a member of an online community of N_2_O users, and based on information provided on their forum, he had been prophylactically taking 1,000 micrograms daily of oral vitamin B_12_ supplementation for the prior three weeks.^3^

The patient was observed to be awake, alert, and fully oriented. He presented via private vehicle to the ED where his initial vitals were notable for mild tachycardia (heart rate of 108 beats per minute [bpm]), but otherwise within normal limits. He was noted to be anxious-appearing and tremulous. His neurologic exam revealed diminished sensation to light touch in the lower extremities below the knees and in the bilateral upper extremities distal to the mid forearms. His sensory deficits worsened distally in all extremities and were symmetric. His cranial nerves were intact. His motor function remained intact and was 5/5 globally. He ambulated with steady gait. His cardiopulmonary exam was unremarkable aside from tachycardia, and there were otherwise no positive findings on his remaining examination.

In the ED, intravenous (IV) access was obtained, and cardiac monitoring was established. He had blood and urine laboratory studies ordered, and neuroimaging was performed. The poison center was consulted. A 1,000 milliliter (mL) normal saline IV fluid bolus was given, as well as 4 milligrams (mg) ondansetron and 2 mg lorazepam IV. He was given 600 mg oral ibuprofen and 650 mg acetaminophen for his headache. A point-of-care glucose measurement was 109 mg per deciliter (dL) (reference range: 70–90 mg/dL). His laboratory studies revealed a complete blood count with differential without significant abnormalities and a comprehensive metabolic panel with a mildly elevated total bilirubin 1.4 mg/dL (0.7–1.2 mg/dL), a slightly elevated aspartate transaminase 37 Units (U) per liter (L) (5–34 U/L), and otherwise normal values. His magnesium level was minimally low at 1.5 mg/dL (1.6–2.6 mg/dL).

**CPC-EM Capsule**

What do we already know about this clinical entity?

*Chronic nitrous oxide (N_2_O) abuse causes neurologic deficits, which may be permanent but improved with cessation of the drug and vitamin B_12_ supplementation*.

What makes this presentation of disease reportable?

*This chronic user of N_2_O prophylactically self-supplemented with vitamin B_12_ but developed neurologic toxicity despite supratherapeutic levels of B_12_*.

What is the major learning point?

*Nitrous oxide abuse creates a functional B_12_ deficiency, which makes prophylactic self-supplementation of B_12_ ineffective in preventing neurotoxicity*.

How might this improve emergency medicine practice?

*Prophylactic self-supplementation of B_12_ is not well-documented in the literature. This case helps broaden our understanding of the impacts of N_2_O on patients*.

A venous blood gas revealed venous pH of 7.45 (7.35–7.43), partial pressure of carbon dioxide of 32 millimeters of mercury (mm Hg) (41–51 mm Hg), partial pressure of oxygen of 32 mm Hg (30–50 mm Hg), and bicarbonate of 24 millimoles (mmol) per L (24–28 mmol/L). A venous lactic acid level was 2.10 mmol/L (0.36–1.25 mmol/L). His total creatine kinase was 159 U/L (30–200 U/L). His ethanol, acetaminophen, and salicylate levels were undetectable. A urine toxicology screen was positive for cannabinoids and cocaine. Thyroid-stimulating hormone level was within normal limits. His methemoglobin level was 0.5% (0.5–1.5%). Vitamin B_12_ level was elevated at greater than 2,000 picograms (pg) per mL (213–816 pg/mL). A computed tomography of the head without IV contrast demonstrated no acute intracranial abnormalities. An electrocardiogram showed a sinus tachycardia with a rate of 104 bpm, without any ischemic changes or notable abnormalities. A high-sensitivity troponin was less than 4 nanograms (ng) per L (less than or equal to 35 ng/L). A two-view chest radiograph demonstrated no acute cardiopulmonary disease.

He was given 2 grams IV magnesium supplementation and observed. Upon re-evaluation, his headache had resolved, and he was no longer tachycardic. He remained hemodynamically stable. He appeared less tremulous but still complained of persistent paresthesias, unchanged from his initial exam. His presentation and laboratory studies were discussed with the toxicology fellow at the Illinois Poison Center, who agreed that his presentation was consistent with chronic N_2_O toxicity and recommended cessation of N_2_O use plus empiric vitamin B_12_ supplementation of 2,000 mcg/day (despite an elevated vitamin B_12_ level on the patient’s presenting labs).

The poison center advised close outpatient follow-up with either neurology or a toxicology clinic and to obtain outpatient magnetic resonance brain imaging. Furthermore, he was asked to monitor for progression or resolution of his symptoms. In this case, the toxicology clinic was located approximately three hours driving distance from the patient’s home, so he preferred to follow up with a local neurology clinic. He was given a referral to neurology for follow-up and a dose of 2,000 mcg of oral vitamin B_12_ was given while in the ED. The prognosis was discussed with the patient, including possible permanent deficits, and he was urged strongly to discontinue N_2_O use and all other illicit drug use. He was given a prescription for oral vitamin B_12_ supplementation of 2,000 mcg/day and agreed to follow up outpatient closely with neurology. Unfortunately, at the time of this case report completion, the patient had not responded to the neurology clinic’s attempts to schedule him for a follow-up evaluation. Neither did he respond to the emergency physician’s follow-up calls.

## DISCUSSION

A literature review of this topic included mostly case reports with the patients presenting after prolonged use and with a clinical syndrome expected of vitamin B_12_ deficiency, including both hematologic and neurologic abnormalities. Most case reports in the current literature were associated with low to normal serum vitamin B_12_ levels.^4^,^6^ There were a few case reports of sensory neuropathies occurring in patients with elevated serum vitamin B_12_ levels, as seen in this case. Our case was unique, given the patient’s involvement in an online community of recreational N_2_O users who recommend prophylactic self-supplementation of B_12_ to avoid adverse effects.^3^

Nitrous oxide toxicity is divided into acute and chronic phases. In the acute phase, users experience sensations of euphoria, hallucinations, and analgesia. The onset of effects occurs within seconds, typically peak at one minute, and completely resolve within several minutes.^2^ The quick onset and resolution of effects makes N_2_O an attractive drug for adolescents and young adults. Most users return to their baseline neurologic and functional status within minutes after single use. Some users with underlying cardiac or lung disease may have complications, including arrhythmias, hypoxia, pneumothorax, or pneumomediastinum.^4^ Rarely, death attributed to arrhythmias, seizure, and asphyxiation has been reported, with postmortem analysis demonstrating pulmonary edema and visceral congestion.^4^

Toxicity related to chronic N_2_O use is believed to be caused by irreversible oxidation of the cobalt ion in vitamin B_12_ leading to impaired conversion of homocysteine to methionine and S-adenosylmethionine in deoxyribonucleic acid (DNA) and myelin synthesis. Vitamin B_12_ exists in the body in two active forms, methylcobalamin and adenosylcobalamin, which act as cofactors for methionine synthetase and methylmalonyl coenzyme A mutase, respectively. Nitrous oxide acts to convert vitamin B_12_ from its active to its inactive form via irreversible oxidation, thus blocking its availability as a cofactor for DNA and myelin synthesis. This leads to demyelination of the central and peripheral nervous systems as well as megaloblastic anemia.^2^

This pathology may present clinically as sensory neuropathy, myeloneuropathy, and subacute combined degeneration.^5^,^6^ While patients with vitamin B_12_ deficiency may be at greater risk of developing neurologic deficits, there are case reports of patients with normal to high serum vitamin B_12_ levels sustaining complications, suggesting the importance of vitamin B_12_ inactivation rather than deficiency that leads to pathogenesis. Some studies have suggested measurement of levels of methylmalonic acid (MMA) in exposures, rather than vitamin B_12_ levels. A rising MMA level signals a functional deficiency of vitamin B_12_, as it is not being normally utilized.^8^,^9^ The patient in this case reported experienced persistent sensory neuropathies and hallucinations despite an elevated vitamin B_12_ level due to prophylactic self-supplementation. This demonstrates a situation in which a normal-to-elevated vitamin B_12_ level may provide false reassurance to clinicians regarding neurologic outcomes. Further research is required to determine whether other endpoints such as folate, homocysteine, or MMA levels may provide more diagnostic reassurance or prognostication of neurologic outcomes.

The patient in this case was lost to follow-up, and it is not known whether he adhered to treatment.

## CONCLUSION

Recreational use of nitric oxide is increasingly prevalent, especially among adolescents and young adults, and it is important to recognize due to the potential for permanent neurologic deficits. Cessation is the mainstay of treatment, but it is important to provide vitamin B_12_ supplementation, even in cases when serum vitamin B_12_ levels are normal or even elevated. These patients require close follow-up with neurology for magnetic resonance imaging and serial evaluations of neurologic function to maximize outcomes. Even when adherent to treatment, patients may never have full neurologic recovery.
